# Horizontal GBR with anorganic equine bone combined with a customized titanium mesh

**DOI:** 10.1002/ccr3.8780

**Published:** 2024-04-23

**Authors:** Francesco Orlando, Simone Foiani, Claudia Dellavia, Daniele Graziano, Danilo Alessio Di Stefano

**Affiliations:** ^1^ Private Practice, Centro Odontoiatrico e Protesico Civitali S.R.L. Milan Italy; ^2^ Dental School Vita‐Salute University IRCCS San Raffaele Milan Italy; ^3^ Department of Biomedical Surgical and Dental Sciences Università Degli Studi di Milano Milan Italy; ^4^ Department of Dentistry Vita‐Salute San Raffaele University Milan Milan Italy

**Keywords:** anorganic bone, equine bone graft, guided bone regeneration, xenograft

## Abstract

This case report describes the fixed rehabilitation of the lower left arch in a patient following an horizontal GBR procedure by means of a customized titanium mesh and a new slow resorption bone substitute of equine origin.

## Graphical abstract

Titanium Mesh application and tissue adaptation.

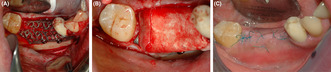

## 1 INTRODUCTION

Guided bone regeneration (GBR) is a surgical technique that allows for the regeneration of several bone defects due to pre‐ or post‐implant bone atrophy, infections, or destructive surgical events. GBR involves the use of a grafting material (e.g., autologous bone, heterologous bone substitutes, and synthetic materials) used as scaffold for the regeneration of the missing bone, and membranes acting as physical barriers to the external environment. This barrier prevents the migration of epithelial and/or connective tissues into the bone defect^1^ as well as potentially pathogenic agents that could cause infections^2^, ^3^ in the newly formed bone.^4^, ^5^, ^6^ Moreover, membranes have the important tasks to contain the grafting material, stabilizing it, and providing a “tent” effect avoiding the premature resorption of the graft due to mechanical stresses.^7^ Different types of membranes can be used in GBR, and they can be classified as resorbable or nonresorbable, accordingly to the different materials they are made of. Nonresorbable membranes include those made of dense‐polytetrafluoroethylene (d‐PTFE), and expanded‐polytetrafluoroethylene (e‐PTFE)^8^ while resorbable membranes include collagen,^9^ pericardium,^10^ and those composed of aliphatic polyesters such as PLA, PGA, and PCL.^11^ In large bone augmentation procedures, where an improved stabilization of the grafting material is required, titanium meshes^12^ can be used to create useful spaces for bone regeneration and to provide an optimized tent‐effect. These meshes have a porous structure with a perforated texture in order to allow blood supply to the underlying tissues.^13^ Often, titanium meshes are covered with resorbable collagen membranes to reduce the penetration of connective tissue cells through the pores.^14^ A recent systematic review suggest that titanium meshes may have a lower risk of postoperative complications compared to nonresorbable membranes.^15^ These results can be explained with the unique feature of titanium mesh in promoting the formation of a pseudo‐periosteum over the bone graft material. This layer is thought to provide a barrier against bacteria.^14^ Moreover, the presence of pores in titanium meshes secures a correct vascularization and nutrients supply to defect.^16^

Although titanium meshes have the necessary rigidity required for the GBR of large bone defects, they have also some disadvantages. In particular, they must be shaped accordingly to the defect, increasing the surgical time of the surgery. Moreover, after the shaping, the titanium mesh may still have some irregularities that may damage the soft tissue, which may affect the final regeneration. A new generation of customized titanium mesh was then developed to overcome such limitations. Indeed, customized titanium mesh will have the correct shape saving surgical time as well as improving the adaptation to the geometry of the defect, resulting in an easier procedure compared to the traditional titanium meshes.^16^, ^17^ Another difference is that the pores of customized titanium meshes are larger compared to the traditional titanium meshes and often a collagen membrane is positioned to further protect the graft. Regarding the grafting materials, numerous types have been used in GBR over the years. These include bioceramic materials such as beta‐tricalcium phosphate (β‐TCP),^18^ hydroxyapatite (HA), biphasic calcium phosphate (BCP), and freeze‐dried bone allograft (FDBA)^19^ as well as heterologous bone substitutes.^6^, ^20^, ^21^, ^22^, ^23^, ^24^ Often, bone regeneration is performed using a mix of heterologous bone and autologous bone chips.^25^ In this case report, we describe a 60‐year‐old male patient with a large horizontal bone defect in the fourth quadrant who was successfully treated using the GBR technique with a titanium mesh and anorganic equine bone, a slow resorbing biomaterial.

## 2 CASE HISTORY

**FIGURE 1:**
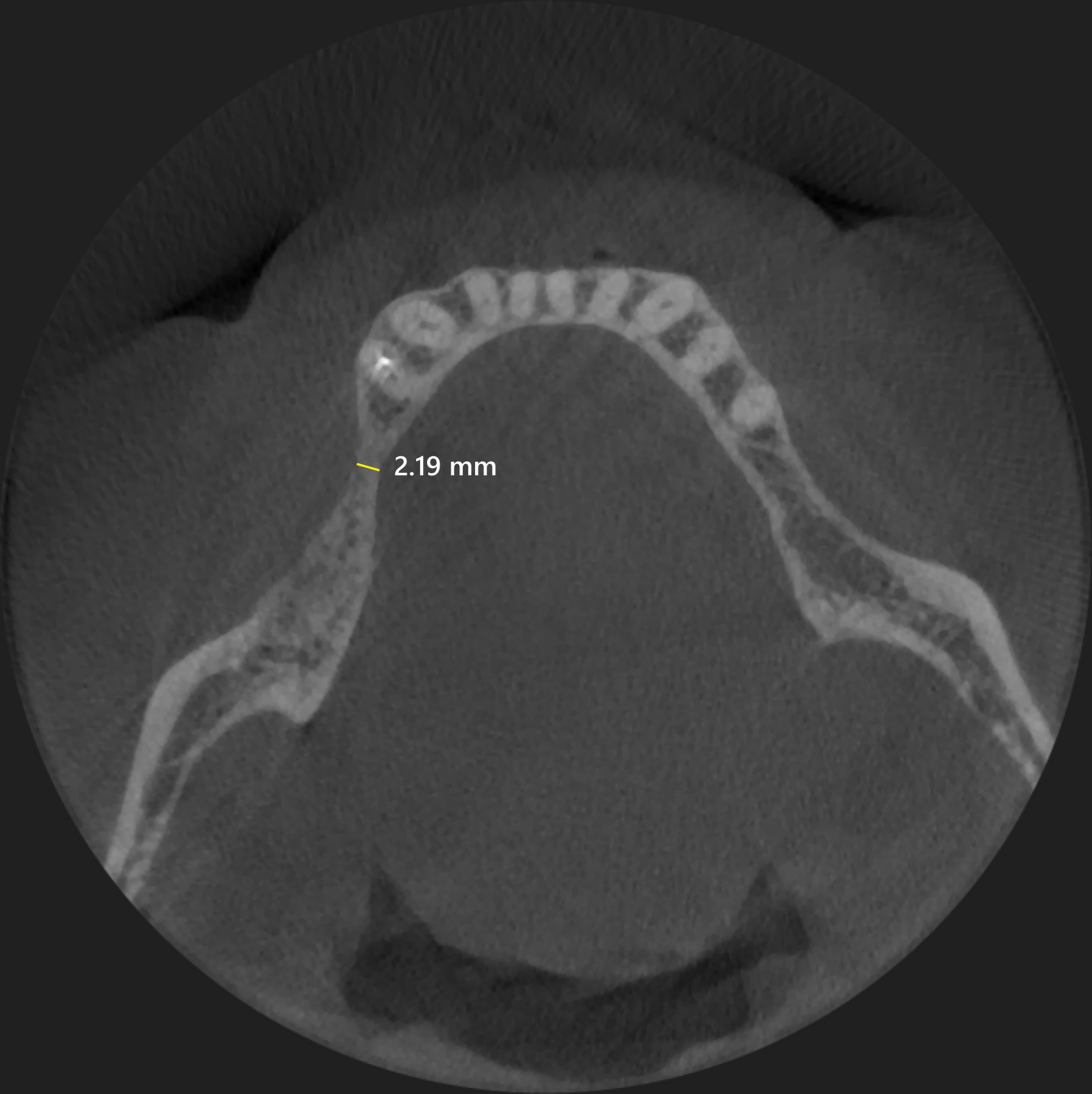
CBCT examination showing the minimal remaining thin crestal ridge (≈2.2 mm) in the fourth anatomical quadrant.

The patient was a nonsmoker 60‐year‐old man, healthy and seeking for a fixed rehabilitation of the lower left arch. The preoperative CBCT scan (Figure 1) revealed a large horizontal defect and a GBR procedure was planned. Due to the thin residual crestal ridge, it was decided to use a 50% mixture of anorganic equine bone (Calcitos®, Bioteck Spa, Arcugnano, Italy) and autologous bone, stabilized with a customized titanium mesh (Yxoss CBR®, ReOss, Filderstadt, Germany). This latter was designed with the CAD‐CAM technology by the manufacturer based on the DICOM files obtained with the CBCT and the planned bone regeneration. The final draft was then digitally approved by the clinician and delivered 2 weeks after.

## 3 METHODS

**FIGURE 2:**
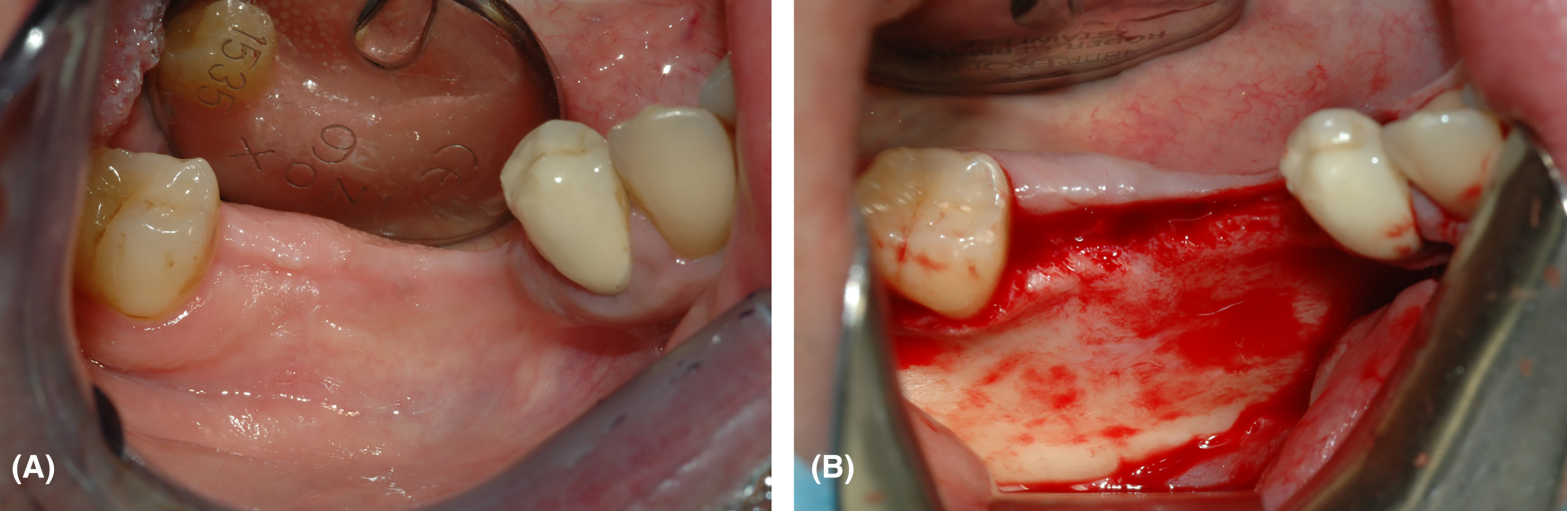
(A) Anatomical extension of the area to be treated related to the fourth anatomical quadrant of the mandible, involving teeth 45, 46, and 47. (B) Access to the bone defect by rising a full‐thickness flap.

**FIGURE 3:**
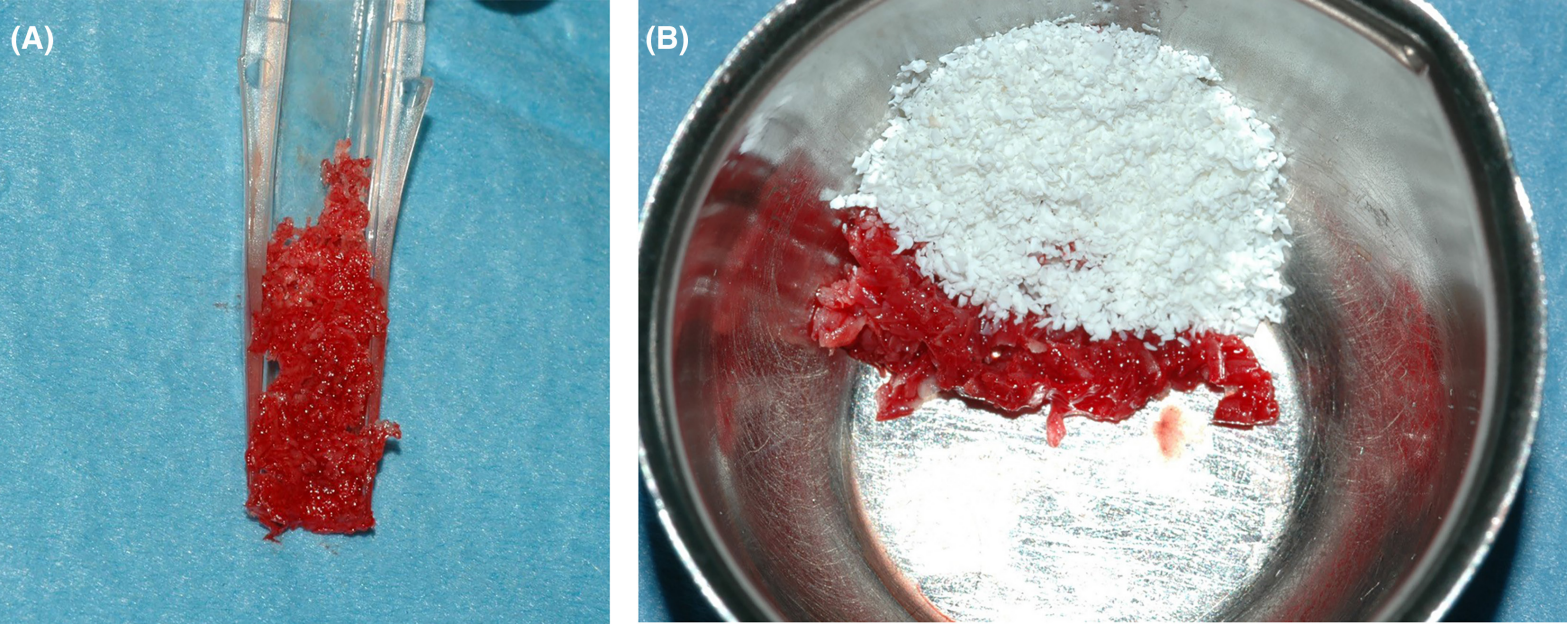
(A) Autologous harvested with the safescraper. (B) Mix 1:1 of the autologous bone with the collagen‐preserved equine bone graft.

**FIGURE 4:**
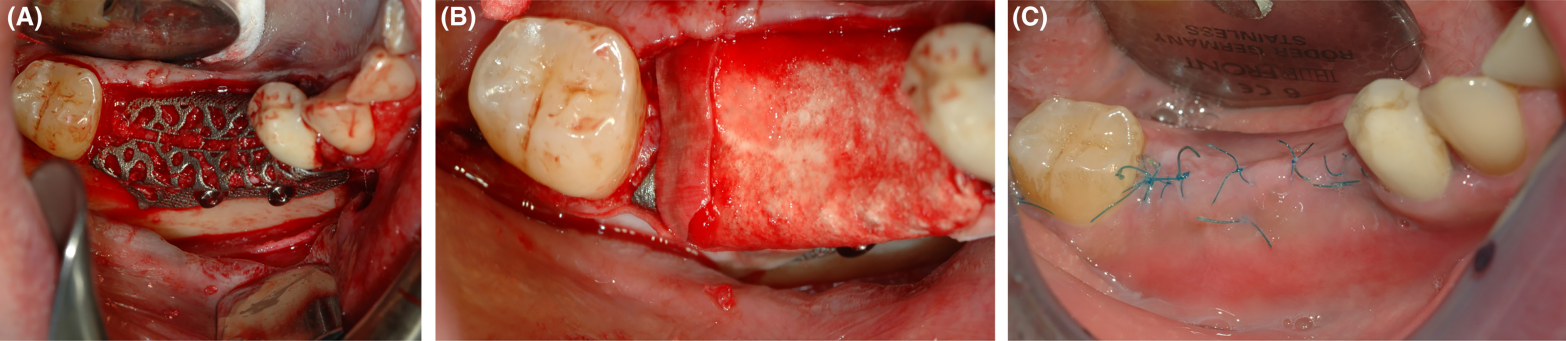
(A) The titanium mesh, prefilled with a mixture of autologous/heterologous bone substitutes, is fixed by using titanium pins (B) The titanium mesh is covered with a resorbable collagen membrane. (C) Flap closure with mattress sutures alternated with single stitches.

The surgery starts with a full thickness flap to access the bone defect (Figure 2). By using a safescraper (Safescraper Twist, Meta Technologies s.r.l., Reggio Emilia, Italy) a few amounts of autologous bone was collected from the grafting site, favoring the natural bleeding of the site. The autologous bone chips were then mixed with a similar amount of anorganic equine bone in a sterile container, and the mix was hydrated with a few drops of sterile saline solution (Figure 3). The mixture was then placed on the bone defect using the mesh itself as an aid. Two titanium pins were used to secure the mesh to the bone (Figure 4A). The mesh was subsequently covered with a resorbable collagen membrane (Biocollagen®, Bioteck Spa, Arcugnano, Italy) (Figure 4B) to further protect the bone graft, and favoring the correct vascularization of the defect site. In order to achieve the closure of the flap, periostal release was performed and horizontal mattress sutures (Monomyd 4‐0/5‐0 Polyamide Monofilament Suture, Butterfly, Cavenago, Italy) alternated with single stitches were placed therefore enabling a secure flap closure and healing by primary intention (Figure 4C).

Prophylactic antibiotics, specifically amoxicillin/clavulanic acid (Augmentin, Glaxo‐SmithKline, Verona, Italy) were administered 2 g 1 h presurgery followed by 1 g doses every 12 h for 8 days. The patient was instructed to continue using chlorhexidine 0.2% mouth rinses (Corsodyl, Glaxo‐SmithKline, Brentfort, United Kingdom) for 2 weeks postsurgery. Nimesulide 100 mg (Aulin, Roche, Milano, Italy) was also given 1 h prior to surgery and then twice daily for a week. The surgical site was numbed using articaine hydrochloride 1% combined with epinephrine at a 1:100,000 ratio (Pierrel, Caserta, Italy).

**FIGURE 5:**
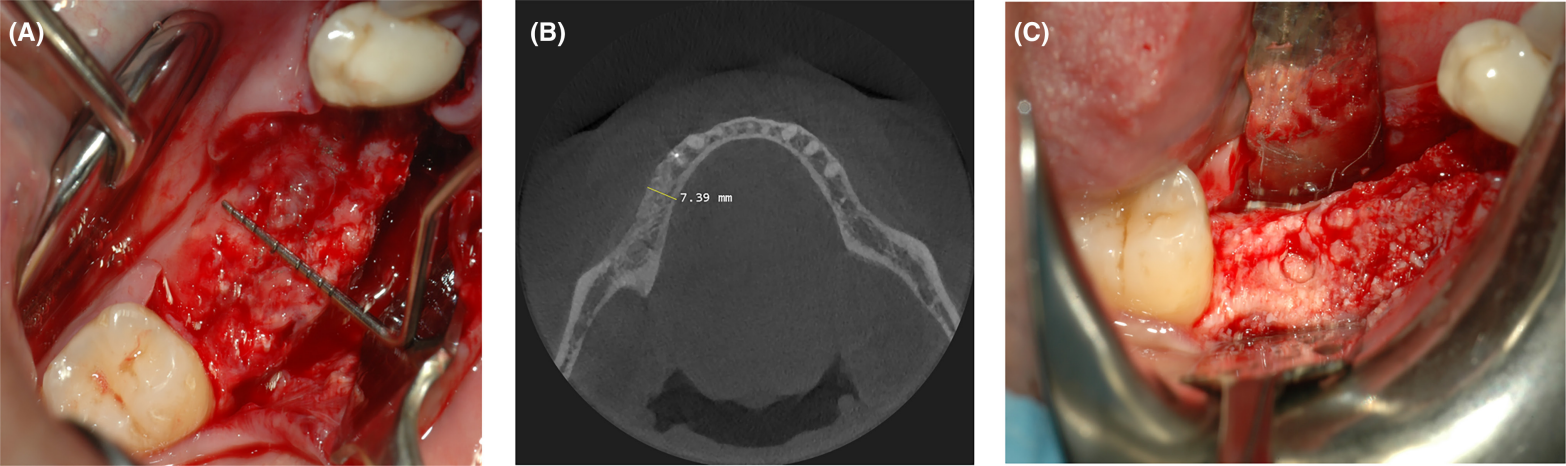
(A) Clinical view of the horizontal increment: the bone appears well vascularized and ready for implant insertion (B) CBCT radiological examination shows a final bone thickness of 7.4 mm, with a 5.2 mm increment compared to the presurgical situation. (C) site of biopsy harvested for subsequent histological analysis.

Nine months after the regenerative surgery, the surgical site was reopened, and the titanium mesh was removed. This allowed for the observation of a substantial amount of newly formed bone, both by clinical inspection (Figure 5A) and through CBCT radiological examination (Figure 5B), which showed a horizontal increase of the bone crest of about 5 mm, with a final crestal width of 7.4 mm, compatible with the insertion of three implants (Dentsply, Xive, Verona, Italy): site 45 3.4 × 13 mm, site 46 3.4 × 11 mm, site 47 3.8 × 9.5 mm.

**FIGURE 6:**
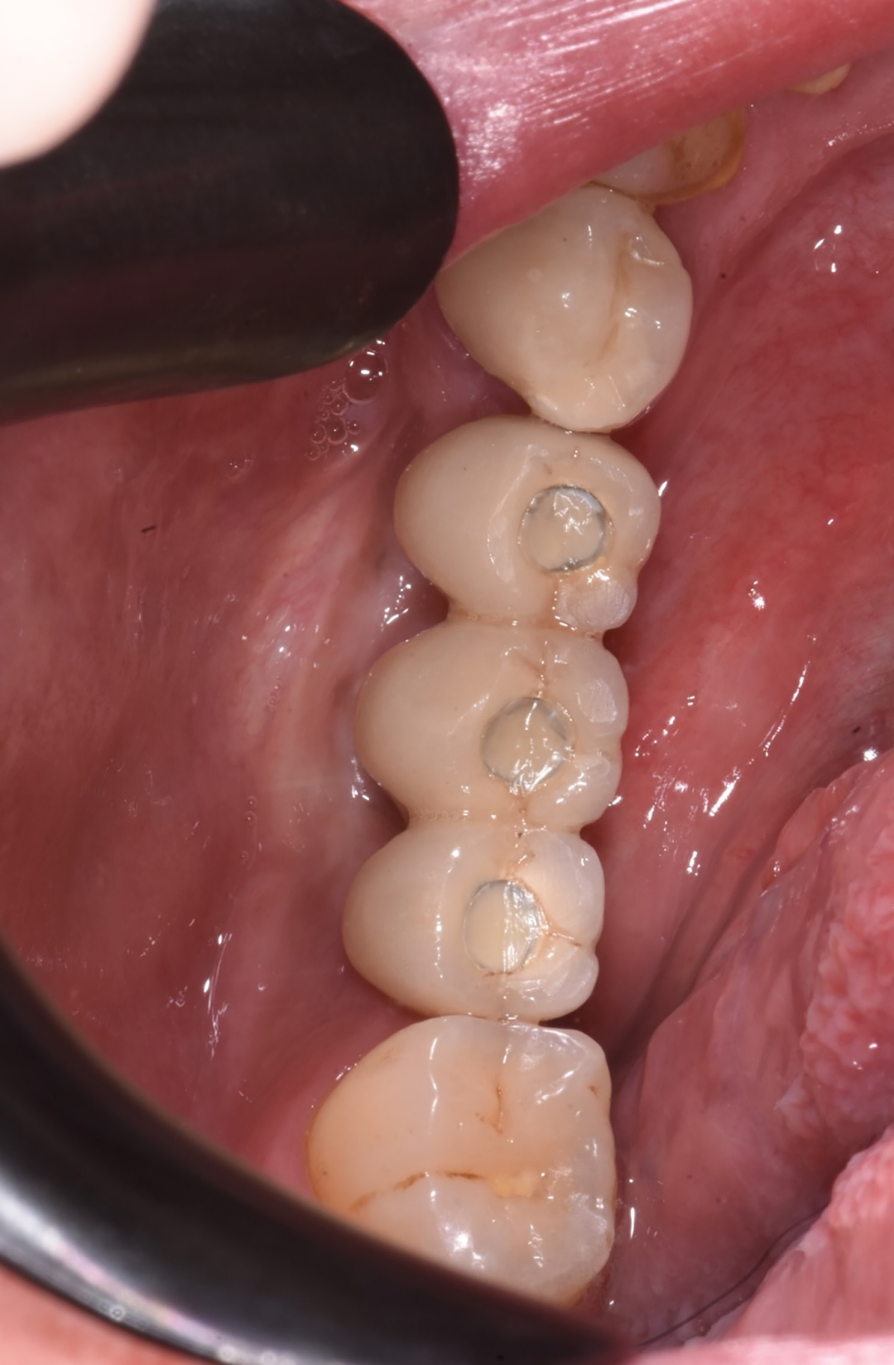
Final prosthesis with a screwed metal‐ceramic structure and the preserved ridge profile.

Along with the preparation of the implant tunnels (Figure 5C), a bone biopsy was harvested using a trephine burr, which was subsequently analyzed histologically and histomorphometrically. The dimensions of the harvested bone core were 3 mm diameter. The sample was fixed in buffered 10% formalin, decalcified by Osteodec (Bio Optica, Milano, Italy), dehydrated in ascending alcohol scale infiltrated, and finally embedded in paraffin (Bio‐Plast, Bio Optica, Milano, Italy). A longitudinal section of 6 μm was obtained in the central portion of the block with a microtome (Leica Biosystems, Milano, Italy) and stained with Carazzi's Hematoxylin and Eosin in order to perform histological and histomorphometric analysis. Images of the samples were captured using high‐resolution digital scanner Aperio CS2 (Leica Biosystems, Milano, Italy) and analyzed with Image Scope software (Leica Biosystems, Milano, Italy). A counting grid was superimposed to the histological section to evaluate the intersection points that fall down on each kind of tissue (regenerated bone, biomaterial, and soft tissue) using the software ImageScope (Leica Biosystems, Milano, Italy). The volume fractions percentage was obtained by the ratio between the intersection points that fall down on each type of tissue and the total intersection points.^26^ Four months later, the provisional prostheses were delivered. After additional 2 months, the final prostheses with a screwed metal‐ceramic structure, was mounted, reproducing the optimal ridge profile with the satisfaction of the patient (Figure 6).

## 4 CONCLUSION AND RESULTS

**FIGURE 7:**
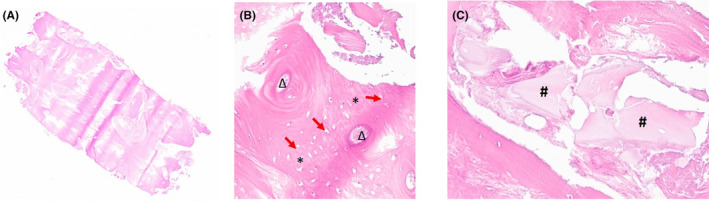
(A) Complete histological section of the sample collected. (B) Detail of the osteon (magnification 300X). Around the osteon, indicated by Delta (Δ), woven bone tissue is arranged (*asterisks) with mature osteocytes whose nuclei are highlighted by the red arrows; (C) Biomaterial residues (magnification 200X). Hashes (#) indicate the presence biomaterial residues fully integrated into the bone matrix.

**FIGURE 8:**
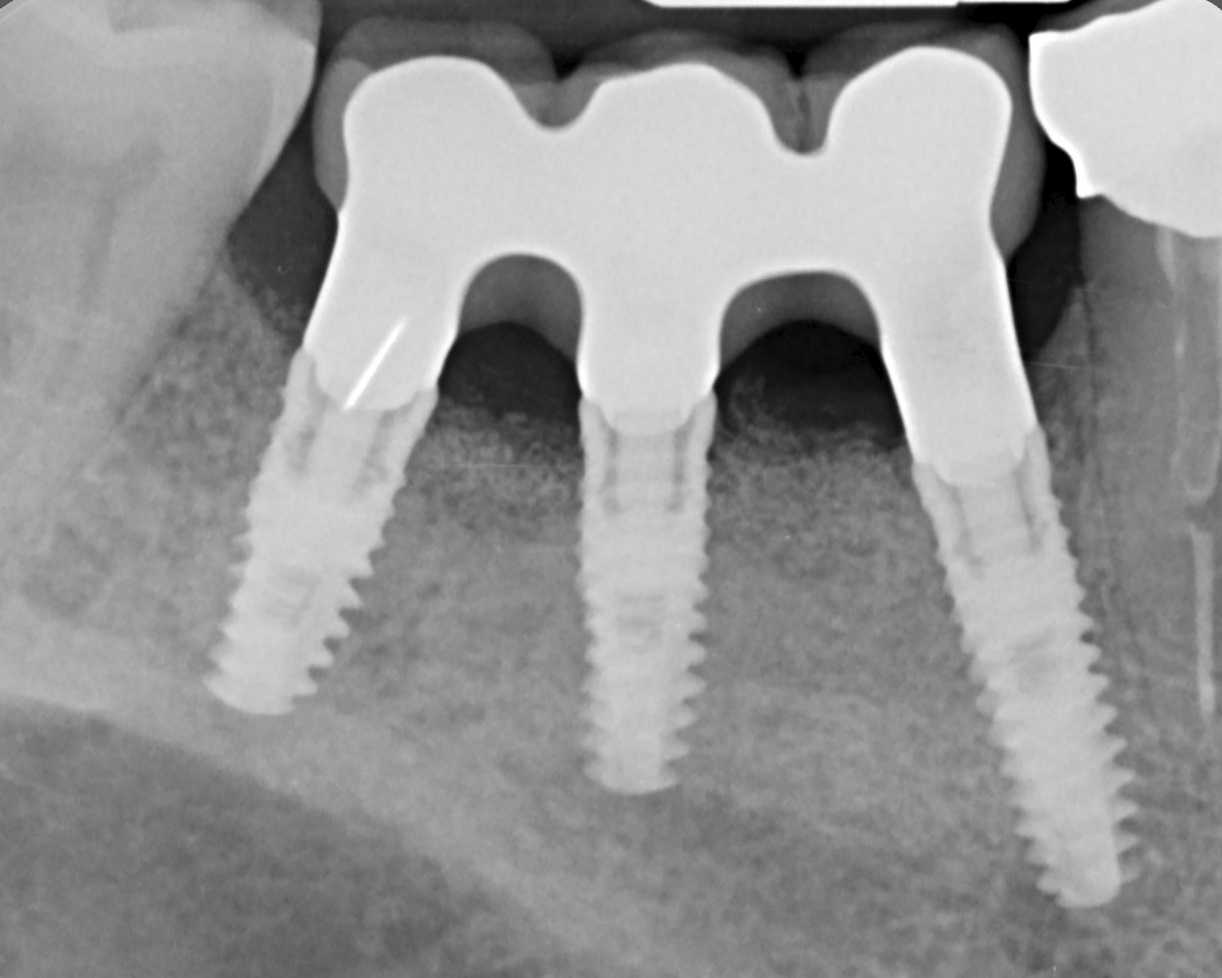
Endoral X‐ray control of the definitive prosthetic with metal‐ceramic structure, showing the optimal regeneration obtained.

In this work, we presented a clinical case of bone regeneration with GBR technique using a thermally treated equine bone substitute mixed with autologous bone and combined with a customized titanium mesh. At 9 months from GBR, CBCT confirmed the presence of sufficient regenerated bone for implant rehabilitation with a horizontal bone augmentation of 5.2 mm. The quality of the bone was evaluated histologically. The examination performed on the bone biopsy, harvested at the same time of implant insertion, showed the absence of inflammatory infiltrate. Histomorphometric measurements showed 79% of newly formed bone, while the residual biomaterial was 2.75%. The remaining 23.08% was constituted by marrow spaces. The bone detected by histological examination appears mature, with the presence of osteons surrounded by lamellar bone (Figure 7). X‐rays examination confirmed the good osseointegration of all the three implants inserted both in the provisional prosthetic phase and in the subsequent definitive prosthetic phase (Figure 8).

The patient did not experience any complications, and it was therefore possible to finalize the placement of three dental implants to complete the prosthetic rehabilitation, with the satisfaction of the patient. The present case report confirmed also the advantages of the customized titanium mesh in large bone defects reconstruction.

## 5 DISCUSSION

One of the most common and complex bone defects to be surgically treated using a regenerative approach is the atrophic ridge, which can occur as a result of dental extractions or long‐term tooth loss. This can lead to a very thin and significantly reduced dimension of the bone ridge.^27^

This is the first clinical case reporting the use of anorganic equine bone in combination with a titanium mesh to perform a large horizontal augmentation. The anorganic equine bone is manufactured through a high temperature process. It has been shown in literature that thermal process reduces osteoclast adherence to the heterologous bones, which results a long‐term resorption time.^28^ Such property can be valuable in large defects, where a prolonged scaffold function is needed to support bone regeneration.

Although resorbable membranes offer a range of clinical advantages, such as to avoid a second surgical re‐entry, titanium meshes have shown greater regenerative performance when applied in the GBR of complex defects. In particular, titanium meshes possess excellent mechanical properties allowing the space‐maintainment and the stability of the bone graft, which are key elements of GBR success.^29^ In addition, titanium meshes reduce the postoperative complications observed with nonresorbable membranes. In the present case report, the limits of traditional titanium meshes related to the difficulties in their modeling, defect adaptation and fixation were overwhelmed by the use of customized titanium mesh obtained with the CAD‐CAM technology. The customized titanium mesh allowed also the reduction of surgical time. In the present case report, the combined use of a CAD‐CAM customized titanium mesh with the equine anorganic bone, a horizontal bone augmentation of 5.2 mm was achieved.

The quality of the bone was evaluated histologically. The examination performed on the bone biopsy, harvested 9 months after the regenerative surgery, showed the absence of inflammatory infiltrate. Histomorphometric measurements showed 79% of newly formed bone, while the residual biomaterial was 2.75%. The remaining 23.08% was constituted by marrow spaces. The bone detected by histological examination appears mature, with the presence of osteons surrounded by lamellar bone (Figure 7).

The high amount of regenerated bone is probably due to the timing of the biopsy harvesting, that is, at an advanced remodeling stage of the biomaterial, and to the use of the titanium mesh, which ensured the best graft stabilization for a favorable advancement of the bone remodeling. X‐rays examination confirmed the good osseointegration of all the three implants inserted both in the provisional prosthetic phase and in the subsequent definitive prosthetic phase (Figure 8).

Anorganic bone of bovine origin have been used over time,^30^, ^31^, ^32^ successfully.

Addis et al^4^ showed that anorganic equine bone exhibits morpho‐structural characteristics similar to those of bovine anorganic bone.^4^ In the study by Poli et al.,^33^ 13 patients undergoing alveolar ridge reconstruction prior to implant placement were treated using a titanium mesh and a combination of autologous bone and deproteinized bovine anorganic bone. In 92.30% of the patients, the postoperative course was uneventful, and all patients achieved a sufficient increase in the thickness of the alveolar crest, allowing for proper placement of the planned dental implants. The implant survival rate at approximately 88 months of follow‐up was excellent, with 100% of cases showing good aesthetic outcomes.

In the study by Pieri et al.,^34^ clinical and radiographic outcomes of implants placed following crestal augmentation using a combination of autologous bone and anorganic bovine bone in a 70:30 ratio with the use of titanium mesh were evaluated. CT scans revealed excellent osseointegration of the implants and an adequate level of crestal augmentation in all patients. Radiographs demonstrated a mean marginal bone loss (MBL)of 0.6 mm at 6 months and approximately 1.3 mm at 2 years postimplantation. Clinically, no pain, sensitivity, or implant mobility were observed 2 years after surgery. In three out of the 44 inserted implants, the MBL was higher than the value indicated in the literature^35^ as a success indicator for implant placement.

In the present case report, the use of a titanium mesh combined with a mixture of autologous bone and anorganic equine bone achieved similar results. In particular, a good amount of newly formed bone was formed and a successful implant rehabilitation was obtained.

## AUTHOR CONTRIBUTIONS

**Francesco Orlando:** Formal analysis; investigation; methodology; validation; visualization; writing – original draft; writing – review and editing. **Simone Foiani:** Software; writing – review and editing. **Claudia Dellavia:** Methodology; software; validation. **Daniele Graziano:** Methodology; software. **Danilo Alessio Di Stefano:** Conceptualization; formal analysis; investigation; methodology; resources; supervision.

## FUNDING INFORMATION

Bioteck Spa will pay APC, if the manuscript will be accepted for publication.

## CONFLICT OF INTEREST STATEMENT

The authors have no competing interests to declare that are relevant to the content of this article.

## ETHICS STATEMENT

All work was carried out in compliance with Ethical Principles for Medical Research Involving Human Subjects outlined in the Helsinki Declaration in 1964 and its later amendments.

## CONSENT

Written informed consent was obtained from the patient to publish this report in accordance with the journal's patient consent policy.

## Data Availability

The data that support the findings of this study are available from the corresponding author upon reasonable request.
